# Characterization of the KRas G12D-inhibitor interactions by differential HDX-MS and molecular dynamics simulations

**DOI:** 10.1016/j.csbj.2025.08.008

**Published:** 2025-08-08

**Authors:** Evgeniy V. Petrotchenko, Brandon Novy, Edith Nagy, Konstantin I. Popov, Jason B. Cross, Roopa Thapar, Christoph H. Borchers

**Affiliations:** a Segal Cancer Proteomics Centre, Lady Davis Institute, Jewish General Hospital, McGill University, Montreal, Quebec, Canada; b Division of Chemical Biology and Medicinal Chemistry, University of North Carolina at Chapel Hill, Chapel Hill, NC, USA; c Institute for Applied Cancer Science, The University of Texas MD Anderson Cancer Center, Houston, TX 77030, USA; d Gerald Bronfman Department of Oncology, Division of Experimental Medicine, Department of Pathology, McGill University, Montreal, Quebec, Canada

**Keywords:** hydrogen-deuterium exchange, mass spectrometry, molecular dynamics, proteindrug binding

## Abstract

Hydrogen-deuterium exchange (HDX) combined with mass spectrometry (MS) is a powerful technique for studying changes in protein structure and dynamics upon ligand binding. Protein-ligand complexes can result in increased protection of peptide-bond amides in HDX indicating protein structure stabilization. We have characterized the interaction of small-molecule inhibitors towards the KRas G12D oncoprotein by intact-protein and bottom-up HDX-MS, in combination with molecular dynamics (MD) simulations. Significant differences in HDX protection were detected upon inhibitor binding in the flexible switch-II pocket of the protein. MD simulations of the free and inhibitor-bound KRas G12D proteins also revealed changes in the hydrogen bond network of backbone amides in the switch-II region upon inhibitor binding, explaining the observed HDX protection changes. We have proposed simple semi-empirical metrics which relate changes in HDX-MS experimental values and observed in MD simulations changes in individual backbone hydrogen-bonds between free- and ligand-bound protein states. This combined HDX-MS and MD approach provides an atomistic picture of changes in the KRas G12D secondary structure upon ligand binding and may be a useful tool for future drug design efforts.

## Highlights

• Combination of HDX-MS and MD simulations for protein-ligand binding characterization.
• Atomic level interpretation of the HDX-MS experimental values.
• Open-closed hydrogen bond status metrics for changes in protection from HDX.

## Graphical Abstract

**Figure:**
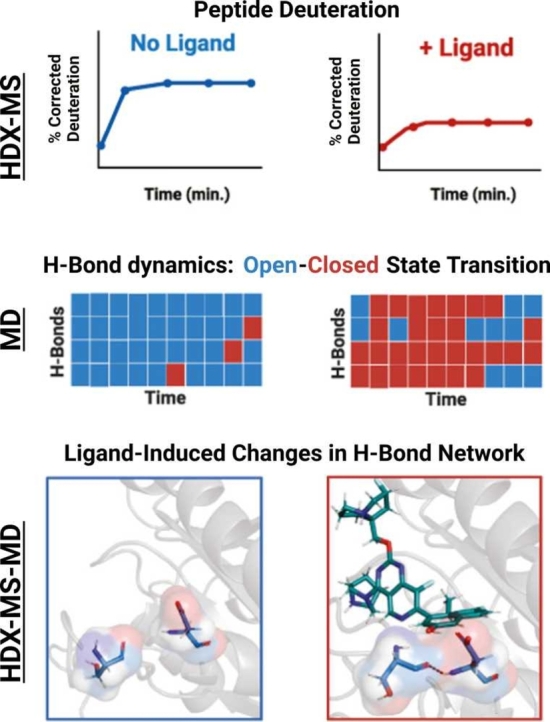

## 1 Introduction

Structural proteomics methods such as limited proteolysis, surface modification, hydrogen-deuterium exchange, and crosslinking, combined with modern mass spectrometry provide unique experimental data for protein structure determination, characterization of conformational changes, and protein-protein and protein-ligand interactions [1]. Historically, these approaches have primarily been used for studying proteins and macromolecular complexes, but they have great potential for studying protein-ligand interactions as well, making them an attractive methodology for drug development applications. HDX-MS, in particular, is an informative technique for studying protein-small molecule interactions [2], [3]. Peptide-bond amide HDX reflects the hydrogen bonding status and the presence of stable secondary structure in proteins. Disordered regions in proteins undergo more efficient exchange and are therefore characterized by higher HDX values. Protein structures can be stabilized by interactions with small-molecule ligands, which can manifest as an increase in protection to HDX upon the formation of the protein-ligand complex. Changes in HDX protection of the target protein upon ligand binding can be measured by intact protein, bottom-up, and top-down MS approaches. Intact protein mass measurements reveal overall stabilization through total deuteration changes, while bottom-up and top-down approaches provide peptide- and residue-specific insights, respectively. These methods help to identify drug binding sites and allosteric conformational changes by revealing changes in deuteration patterns upon ligand binding. [4]. MD simulations provide insights into protein structure and dynamics, and can describe the behaviour of the backbone amide hydrogen bonds (H-bonds) that were measured by HDX-MS [5]. Measuring the differential HDX levels between the free and ligand-bound protein states can, in turn, characterize conformational changes upon ligand binding and provide insights into the mechanisms of ligand binding [6]. A combination of such analyses with MD simulations can facilitate modeling of protein-ligand complexes [7] and can pinpoint the locations of the affected H-bonds, which is critical for the interpretation of HDX-MS data in the drug development and optimization process. Previously, we proposed a simplified empirical approach for determining the opening frequencies of the backbone amide H-bond-forming donor-acceptor pairs along the MD trajectories to assist in interpretation of the mass spectrometry-derived HDX values [8], [9]. Here, we extended this methodology to describe changes in protein H-bond networks upon inhibitor binding, and to identify structural features in drug-like ligands that drive protein conformational changes.

KRas G12D is one of the most common oncogenic drivers in human cancers. The high prevalence and poor prognosis associated with KRas G12D mutations in various cancers makes it an attractive target for drug design. KRas switch II is a key regulatory region that controls the dynamic transition between the protein’s active and inactive states [10]. Targeting this region is a major focus in drug discovery, offering a strategy for the development of small-molecule inhibitors that disrupt KRas signaling. In this work, we have characterized the interaction of inhibitors that bind to the flexible switch-II pocket of mutant KRas G12D proteins, using differential intact-protein and bottom-up HDX-MS in combination with MD simulations.

HDX-MS analysis has previously been successfully used to describe the covalent inhibitors of the KRas G12C [11]. Recently, MRTX1133, a potent and highly selective KRas G12D inhibitor was reported [12], [13]. Several compounds belonging to the MRTX1133 pyrido[4,3-*d*]pyrimidine scaffold that bind KRas G12D with a range of affinities have also been disclosed (US Patents WO2021041671, WO2022031678, WO2022132200). In this study, we investigated the binding of representative small molecules to the KRas G12D protein using a combination HDX-MS and MD simulations. Our goal was to explore the complementary strengths of these two techniques for characterizing protein–ligand interactions, a critical aspect of small molecule drug discovery. HDX-MS provides insights into changes in backbone amide hydrogen bonding upon ligand binding, while MD simulations offer atomistic, per-residue mechanistic details that enhance the interpretation of the HDX data. By comparing the ligand-bound and ligand-free states of KRas G12D, this integrated approach enables a detailed characterization of ligand-induced protein dynamics and supports the development of conformationally selective small molecule modulators.

## 2 Experimental procedures

### 2.1 Protein samples

Recombinant KRas G12D C118S (residues 1–169) protein was expressed in *E.coli* BL21 (DE3) RIL cells using a pET28a vector, and was purified to homogeneity using standard nickel-affinity and size-exclusion chromatographies. The C118S mutation was previously shown to have no effect on the KRas G12D structure, but it enhances the stability of Ras proteins [14]. 10 mM DMSO stock solutions of compounds were stored until use at −20°C.

### 2.2 Inhibitor compounds

Inhibitor compounds were synthesized at the Institute for Applied Cancer Science of the University of Texas MD Anderson Cancer Center. Binding affinities were determined by surface plasmon resonance.

### 2.3 Differential hydrogen-deuterium exchange

For the intact-protein HDX analyses, 2 µL of free or ligand-bound KRas G12D protein samples were mixed with 8 µL of H_2_O or D_2_O (1:4 v:v), incubated for varying time intervals at room temperature (23 °C), quenched with 10 µL (1:1 v:v) of 0.2 % FA in H_2_O, and 10 µL was immediately injected for LC-MS analysis. LC-MS analysis was performed on a TripleTOF 6600 mass spectrometer (Sciex) interfaced with a Nexera LC system (Shimadzu). Chromatography was performed using short 3-min gradients of 0–100 % acetonitrile in 0.1 % FA at 200 µL/min flow rate, using an Agilent C_18_ 300 Å, 3 μm particle size, 2.1 mm i.d. x 50 mm long column, cooled to 0 °C in ice bath, MS1 spectra were acquired over the mass range from 100 to 2000 Da. Analyses were performed in triplicate. Spectra were deconvoluted using PeakView and BioToolKit software (Sciex). Deuteration of the KRas G12D protein was determined based on the mass shift from the mass of the non-deuterated KRas G12D protein. In-exchange was estimated from the deuteration at 0 s, back exchange was estimated from fully deuterated horse myoglobin as ∼30 %.

For bottom-up HDX analyses, 2 μL of free or ligand-bound KRas G12D protein samples were mixed with 8 µL of H_2_O or D_2_O (1:4 v:v), incubated for varying time intervals (0, 20, 40, 80 and 160 s), quenched with 10 µL (1:1 v:v) of 0.2 % FA in H_2_O, supplemented with 2 µL of freshly prepared 1 mg/mL pepsin solution in H_2_O, placed in an autosampler for ∼30 s at 10 °C, and 10 µL was immediately injected for LC-MS analysis. Peptic peptides from the KRas G12D protein were identified from information-dependent acquisition (IDA) LC-MS/MS analysis of the protein in water. Data were analyzed by Protein Pilot (Sciex) software. Chromatography was performed as described above. Spectra were analyzed by PeakView software (Sciex). Deuteration of the peptides was determined using MassSpecStudio software package [15]. Differential bottom-up HDX protection values were visualized using a blue-white-red palette with red set as the maximum observed Δ% of the corrected deuteration values of the peptic peptides.

### 2.4 MD simulations and data analysis

The crystal structure of KRas G12D in complex with MRTX1133 (PDB ID: 7RPZ) was prepared using Schrödinger’s Protein Preparation Wizard with default settings, and all crystallographic water molecules were removed during grid generation for molecular docking [16], [17]. The ligand structure for MRTX1133 was prepared using LigPrep to sample possible protonation and tautomeric states during the docking process [18]. Docking was performed using Glide SP, and the top-scoring pose was selected for downstream molecular dynamics simulations [19]. To validate the docking protocol, we confirmed that the docked pose of MRTX1133 aligned within 1.0 Å RMSD of its crystallographic conformation, demonstrating high reliability in pose prediction. This validation step is shown in Supplementary Figure S1. MD simulations were carried out using GROMACS 2020.3 on the UNC high-performance computing cluster equipped with Nvidia V100 GPUs. Systems were simulated in triplicate for 500 ns each under constant temperature (300 K) and pressure (1 atm), using explicit TIP3P water solvent and 0.15 M NaCl to mimic physiological ionic strength. The CHARMM force field was used for protein parameterization, while ligand parameters were generated using CHARMM-compatible force fields via the CGenFF and SwissParam tools [20], [21], [22], [23]. MD trajectories were processed using the MDTraj package [24] in Python with a timestep of 1 ns for a total of 500 frames. To estimate the changes in the secondary structure, the dynamics of the H-bonds along the trajectory were analyzed by monitoring the distances between backbone H-bond-forming donor-acceptor atom pairs. H-bonds were identified using the Baker-Hubbard algorithm [25], which applies a donor-acceptor distance cutoff of 2.5 Å and a minimum donor-hydrogen-acceptor angle of 120°. The total lifetime of H-bonds were visualized to identify dynamic fluctuations or stability changes, including transient flickering or ligand-induced stabilization. To quantify protection factors and annotate structural features, we aggregated the donor occupancy across replicates. H-bonds present in less than 40 % of the 500-nanosecond trajectory in either simulation set were filtered out, as these transient interactions would have undergone deuterium exchange and were not relevant for analysis. H-bonds exhibiting significant changes in protection factors were identified using a cutoff of + -20 % in occupancy difference between the simulation sets.

## 3 Results and discussion

We used HDX-MS analysis to characterize the binding of the KRas G12D protein with five inhibitors. We observed increased protection from HDX for these compounds. MD simulations of free and ligand-bound proteins corroborated the results, offering an atomic-level view of secondary structure changes in KRas G12D that drive the observed HDX shifts upon ligand binding. Here, we highlight analysis of the representative KRas G12D - MRTX1133 complex to clearly illustrate the utility of combination of HDX-MS and MD simulations for characterizing protein dynamics upon ligand binding.

### 3.1 Changes in HDX protection of KRas G12D protein upon ligand binding

Differential intact and bottom-up HDX-LC-MS analyses were performed to identify conformational changes of the KRas G12D protein upon inhibitor binding. For differential HDX analysis, free and ligand-bound protein samples were exposed to D_2_O solution for varying time intervals, and the deuteration levels were measured by mass spectrometry. Differences in total deuteration between free and ligand-bound KRas G12D reflect the overall stabilization of its secondary structure upon complex formation. Analysis of the differences in deuteration of the peptides provides the locations of differentially protected regions upon complex formation and therefore can reveal the location of the inhibitor-binding site.

**Fig. 1:**
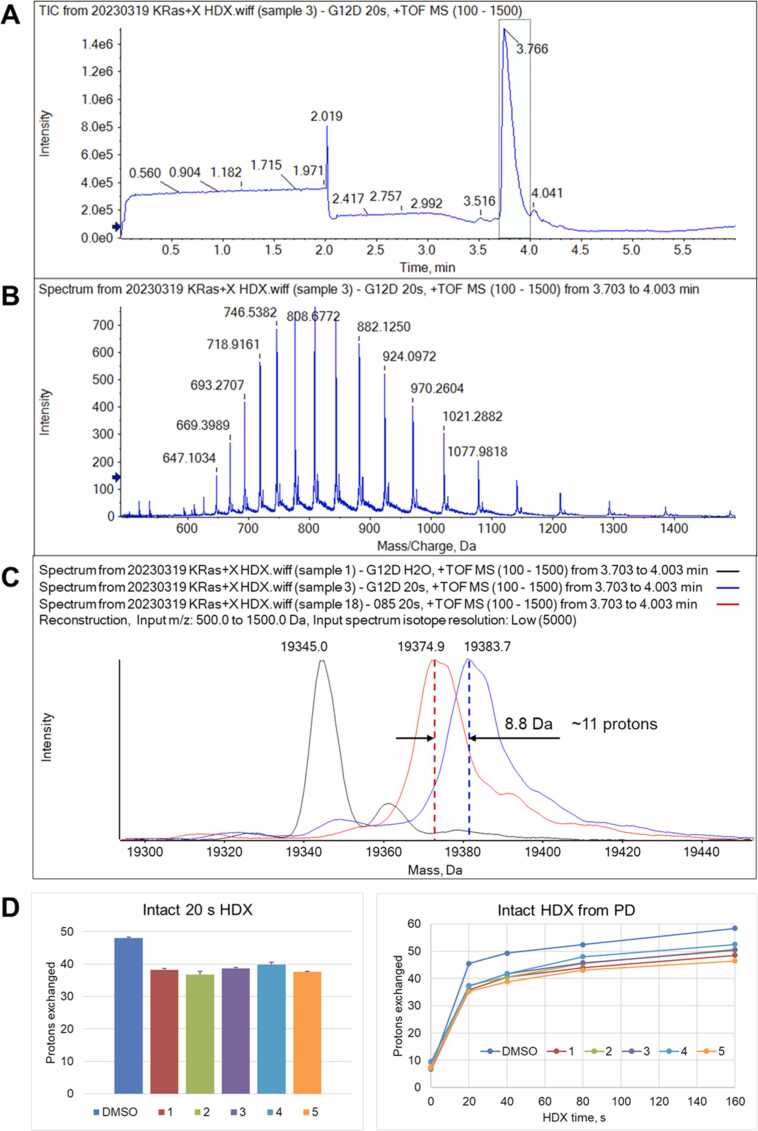
Intact protein mass HDX-LC-MS analysis of the KRas G12D ligand-protein interactions**. A.** Representative LC-MS total ion chromatogram of the KRas G12D deuterated sample (20 s exchange time). **B.** Representative intact-protein cumulative mass spectrum (i.e., accumulated over the entire chromatographic peak for the protein, as highlighted in panel A). **C.** Deconvoluted cumulative mass spectra of non-deuterated sample (black), deuterated (20 s exchange time) ligand-free (blue) and MRTX1133 ligand-bound (red) KRas G12D protein. The 8.8 Da difference corresponds to a change in protection of ∼11 protons (corrected for 80 % D_2_O used). **D.** Changes in KRas G12 HDX protection for different compounds. Left, the intact mass 20 s HDX (n = 3); right, kinetic plots from the intact-mass HDX spectra.

Intact HDX analysis of free and inhibitor-bound Kras G12D samples revealed a significant increase in protection of ∼11 protons upon formation of the complexes, at the 20 s exchange time (Fig. 1). Previously, we have shown that this exchange time provides a good correlation between the measured deuteration values and the number of the H-bonds in the crystal structure of a folded protein [26]. The observed value can serve as a good estimate of the number of H-bond-forming donor-acceptor atom pairs undergoing changes upon protein-ligand complex formation. This analysis is quite precise (%CV<3) and fast, and can be adapted as a screening method for lead drug-like candidates. In the current study, we observed some subtle differences in differential exchange for compounds of differing affinities (Fig. 1D).

**Fig. 2:**
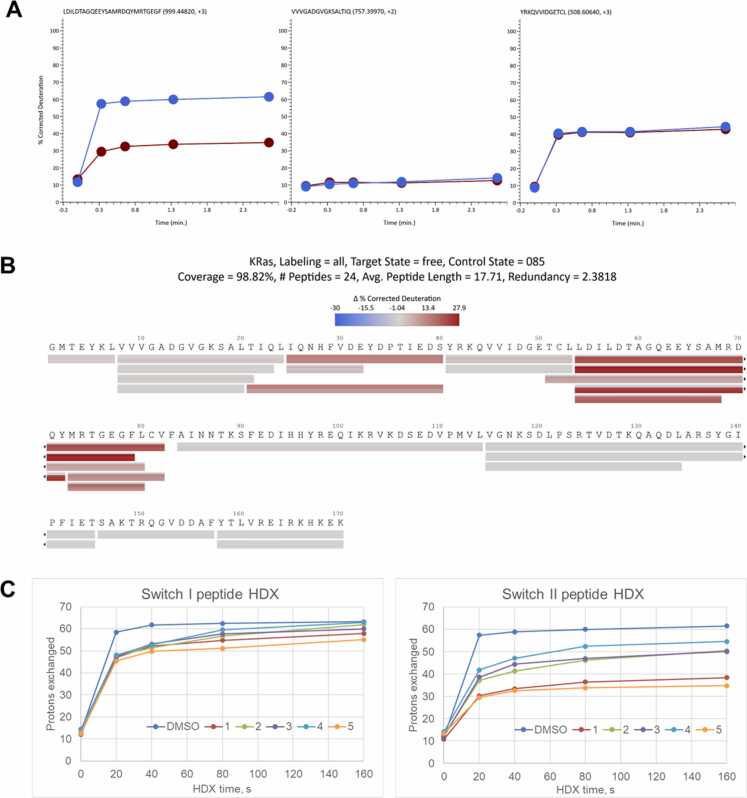
Bottom-up HDX-LC-MS analysis of the KRas G12 protein – ligand interactions**. A.** Representative kinetic plots of KRas G12 peptic peptides HDX. From left to right: a differentially protected peptide upon the complex formation peptide, similarly protected slow-exchanging and fast-exchanging peptide. Blue, free protein; red, MRTX1133 ligand-bound protein. **B.** Differences in deuteration of the peptic KRas G12D peptides between the ligand-free and MRTX1133-bound KRas G12D samples. Values are presented in a blue-white-red palette, with red representing higher protection values in KRas G12D-ligand sample compared to the free KRas G12D sample. Bars represent peptides. Horizontal slices of bars from top to bottom correspond to 0, 20, 40, 80 and 160 s HDX time points. Switch I region – 30–40, switch II region - 60–70. **C.** Changes in KRas G12D HDX protection for different compounds. Left, kinetic plots of switch I peptide; right, kinetic plots of switch II peptide.

Differential bottom-up HDX analysis of free and ligand-bound KRas G12D protein samples also revealed a few peptides exhibiting increased protection against exchange upon ligand-KRas G12D complex formation (Fig. 2). Visualization of the observed changes on the three-dimensional structures of the KRas G12D allowed us to locate the regions which showed an increase in protection upon inhibitor binding. The regions that exhibited the highest changes involved loops which were flanked by secondary-structure elements. These changes in protection were ligand specific, and, notably, these differences were similar for both bottom-up and intact HDX analyses. The extent of the change in protection upon complex formation somewhat correlated with the inhibitor binding affinities [27]. These observed changes were not due to differences in occupancy, as all inhibitors used in the HDX experiment saturated the protein by over ∼97 % under the given conditions (Figure S2). The changes in HDX upon ligand binding were mainly detected in the peptides spanning the switch II region. This is consistent with the location of the inhibitor binding site in the cleft between the switch II loop and the core of the protein, as was observed in the crystal structure of the KRas G12D-MRTX1133 complex (PDB ID: 7RPZ). The observed changes in HDX protection upon protein-inhibitor complex formation therefore suggest an increase of secondary structure in the switch II loop.

### 3.2 Molecular dynamics interpretation of the experimentally observed HDX values

HDX values measure the exchange of the proton from the nitrogen atom of the backbone amide with a deuteron from the solvent. MD simulations in an aqueous environment allow the tracking of the behavior of each hydrogen atom in the protein throughout the trajectory. A common simplifying assumption is that hydrogen-bonded backbone amide protons either do not exchange with solvent protons or do so at a significantly slower rate than non-bonded hydrogen atoms. The extent of this exchange will depend on the fraction of time a given amide hydrogen is in a bonded versus a free state. By monitoring the states of selected peptide bond amide hydrogens along the simulation trajectory, we can quantify the number of frames at which they exist in a free or bonded state. Hydrogens that spend more frames in the free state correspond to highly exchangeable protons, whereas those that remain bonded for a greater number of frames correspond to protected hydrogens [5].

Previously, we showed a correlation between frequencies of H-bond opening for peptide bond amides with observed HDX values [8]. Here, we have extended this semi-empirical metric and applied it to the characterization of experimentally determined bottom-up HDX values. The total number of the backbone amides potentially forming H-bonds was estimated from the crystal structure of the KRas G12D - MRTX1133 complex (PDB ID: 7RPZ) as 102 (∼60 % of the total number, 170). In free KRas G12D, 69 amides were estimated to be deuterated at a 20 s exchange time, which corresponds rather well to the number of non-bonded (based on crystal structure) backbone amide protons. Ligand binding results show an increase in the protection of ∼11 protons at a 20 s exchange time (Fig. 1).

**Fig. 3:**
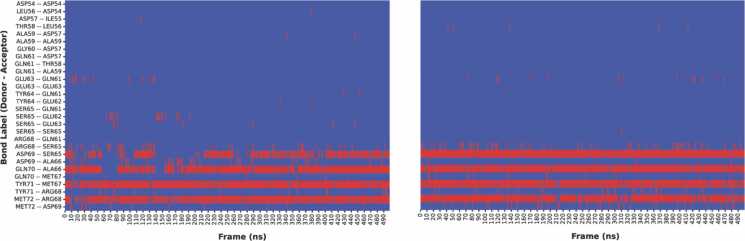
Changes in backbone amide hydrogen bonding in the switch II region, as observed in MD trajectories upon KRas G12 protein-MRTX1133 ligand binding**.** Transient hydrogen bonds in ligand-free state (left) are stabilized upon ligand binding (right), as illustrated by increased number of frames with occupied H-bonds (shown in red).

MD trajectory analysis of both the free and ligand-bound states revealed that several H-bonds in the switch II region of KRAS G12D were stabilized in the presence of MRTX1133 (Figure 3, S3).

**Fig. 4:**
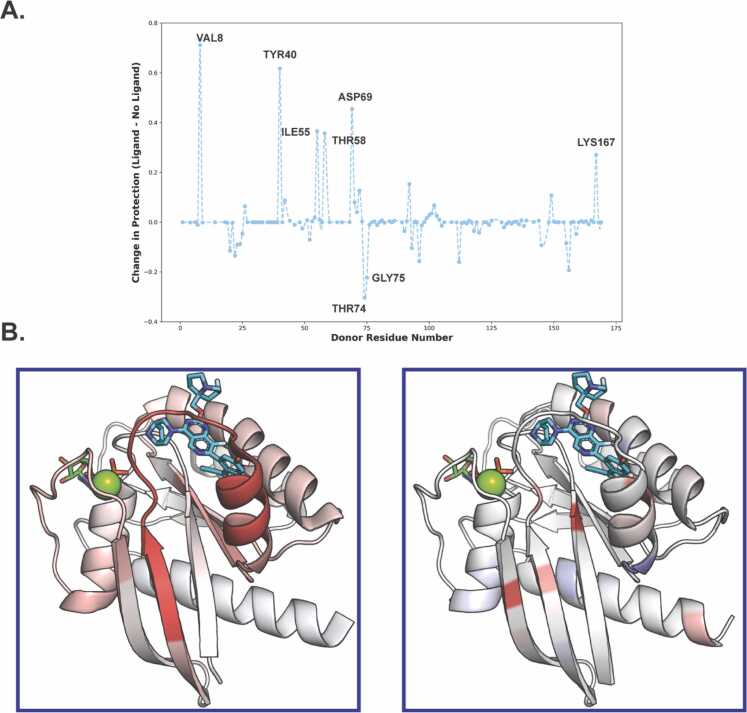
Changes in HDX protection upon KRas G12 protein-ligand binding from HDX-MS and MD approaches**. A.** H-bonds above zero show increased protection upon MRTX1133 ligand binding. To include H-bonds observed in simulations but below the lifetime threshold, their donor residues were imputed to zero (91 H-bonds above cutoff in either set, 131 total donors displayed). **B.** Visualization of deuteration differences (left) and H-bond occupancy from MD simulations (right) between free and MRTX1133-bound KRas G12D. The values are plotted on the KRas G12D- MRTX1133 3D structure (PDB: 7RPZ) using a blue-white-red palette, with red indicating higher protection values and stable H-bonds in the KRas G12D-ligand complex compared to ligand-free KRas G12D.

**Fig. 5:**
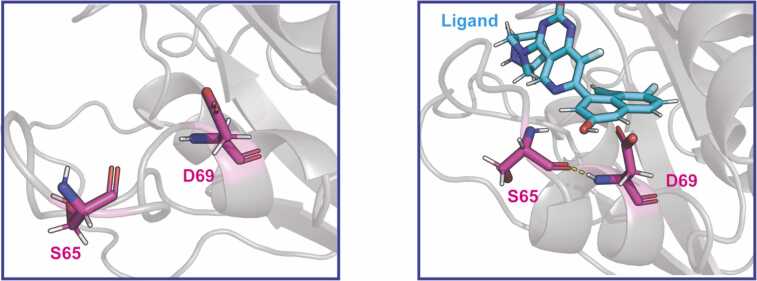
Representative example of hydrogen bond formation within the switch II region upon KRas G12-MRTX1133 ligand binding**.** Left, ligand-free state; right, ligand-bound state.

For the entire protein, we identified a total of 91 backbone amide H-bonds (Figure 4A, S4), which agrees well with H-bond counts in HDX-MS data and crystal structure estimates. All of the H-bonds we identified were mapped onto the protein structure on a per-residue basis and are displayed in Fig. 4B. Eight H-bonds displayed substantial shifts in protection (Fig. 4A) across our replicate simulations. Notably, our analysis confirms that most of the significant changes in protection are located within the switch II region where ligand binding increased the protection. These results align well with the experimental HDX-MS data. For example, the Asp69-Ser65 H-bond forms upon residue coordination with the ligand’s hydroxyl group stabilizing the switch II loop. In the absence of the ligand, this region becomes more dynamic, exhibiting reduced H-bond coordination. (Fig. 3, Fig. 5). The observed correlation between changes in HDX protection upon ligand binding and the affinities of the binding (Figure S2C) can be explained by differences in the numbers of ligand-protein atom-atom interactions formed and the mobility of the ligands at the binding site (discussed in detail in [28]). Higher-affinity ligands produce stiffer ligand-protein complex structures, resulting in limited mobility of both the core protein and the switch II loop, which, in turn, can be detected by both HDX and LiP-MS. In general, ligand binding stabilizes the switch II residues and restricts loop movement, increasing the chance that H-bonds within the loop will form and remain intact. This leads to a higher degree of protection from proton exchange, as measured by HDX-MS (Fig. 5).

The combination of the HDX-MS and MD analyses shown here greatly increases the analytical power for the characterization of ligand-protein binding. Using our simple metric of individual H-bond status, we obtained remarkably good agreement between the MD simulation data and the experimentally observed changes in HDX protection upon ligand binding (Fig. 4A). The incorporation of the MD simulation data into readout of HDX-MS greatly enhances the resolution of this approach. In bottom-up HDX-MS, resolution is usually limited to the length of the peptides produced during the analysis. For example, in our case, the main changes in protection were detected in the 54–79 switch II peptic peptide, while interpretation of the observed changes in H-bonding network from MD simulation enabled us to narrow it down to a few single residues (Fig. 4B). For drug development efforts it is invaluable not to only observe stabilization of the overall protein structure of protein regions, but also to understand which particular H-bonds undergo changes upon ligand binding. Applying this approach -- in combination with atomistic characterization of the ligand dynamics at the drug binding site [28] -- to selected representative compounds from the library during drug development may allow targeted intelligent ligand structure design or “hit” selection.

Overall, the combined HDX-MS and MD analyses explain the experimentally observed HDX protection changes upon inhibitor binding and provide atomic-level insights into the mechanisms of protein conformational changes.

## 4 Conclusions

We have characterized ligand-induced stabilization of the protein secondary structure by HDX-MS-MD. We propose semi-empirical metrics for observed experimental HDX values based on H-bonding status from MD trajectories. The analysis provides atomistic details of the H-bond network and interpretation of the experimental data and can be generally applicable for other protein-ligand systems.

## CRediT authorship contribution statement

**Evgeniy V. Petrotchenko:** Writing – review & editing, Writing – original draft, Methodology, Investigation, Formal analysis, Conceptualization. **Edith Nagy:** Resources. **Brandon Novy:** Writing – review & editing, Writing – original draft, Visualization, Investigation, Formal analysis. **Jason B. Cross:** Writing – review & editing. **Konstantin I. Popov:** Writing – review & editing, Supervision, Methodology. **Christoph H. Borchers:** Writing – review & editing, Funding acquisition. **Roopa Thapar:** Writing – review & editing.

## Declaration of Competing Interest

None.
