# Supplementary material for: Characterization of the KRas G12D-inhibitor interactions by differential HDX-MS and molecular dynamics simulations
Source: Comput Struct Biotechnol J. 2025 Aug 8;27:3618–24. doi: 10.1016/j.csbj.2025.08.008 (PMC12362407; doi:10.1016/j.csbj.2025.08.008)
Supplement: Supplementary file 1 — Supplementary material [file mmc1.docx]

**Characterization of the KRas G12D-inhibitor interactions by differential HDX-MS and molecular dynamics simulations**

Evgeniy V. Petrotchenko^1‡^, Brandon Novy^2‡^, Edith Nagy^3^, Konstantin I. Popov^2^, Jason B. Cross^3^, Roopa Thapar^3^, Christoph H. Borchers^1,4,5,6*^

^‡^ These authors contributed equally

^1^Segal Cancer Proteomics Centre, Lady Davis Institute, Jewish General Hospital, McGill University, Montreal, Quebec, Canada

^2^Division of Chemical Biology and Medicinal Chemistry, University of North Carolina at Chapel Hill, Chapel Hill, North Carolina

^3^Institute for Applied Cancer Science, The University of Texas MD Anderson Cancer Center, Houston, TX 77030, USA

^4^Gerald Bronfman Department of Oncology, ^5^Division of Experimental Medicine, ^6^Department of Pathology, McGill University, Montreal, Quebec, Canada

*Corresponding author:

Christoph H Borchers, Ph.D.

Segal Cancer Proteomics Centre, Lady Davis Institute for Medical Research,

Jewish General Hospital, McGill University

Montréal, Quebec, H3T 1E2, Canada

[christoph.borchers@mcgill.ca](mailto:christoph.borchers@mcgill.ca)

Tel.: +1 514-340-8222 ext.27886

**Supplementary data**

Figure S1. Overlay of docked and experimental MRTX-1133 ligand structures.

Figure S2. Compounds used in the study.

Figure S3. Hydrogen bonding status of the backbone amides in the KRas G12D switch II region in the ligand-free and ligand-bound states.

Figure S4. Hydrogen bonding status of the backbone amides of the KRas G12D protein in the ligand-free and ligand-bound states.

**
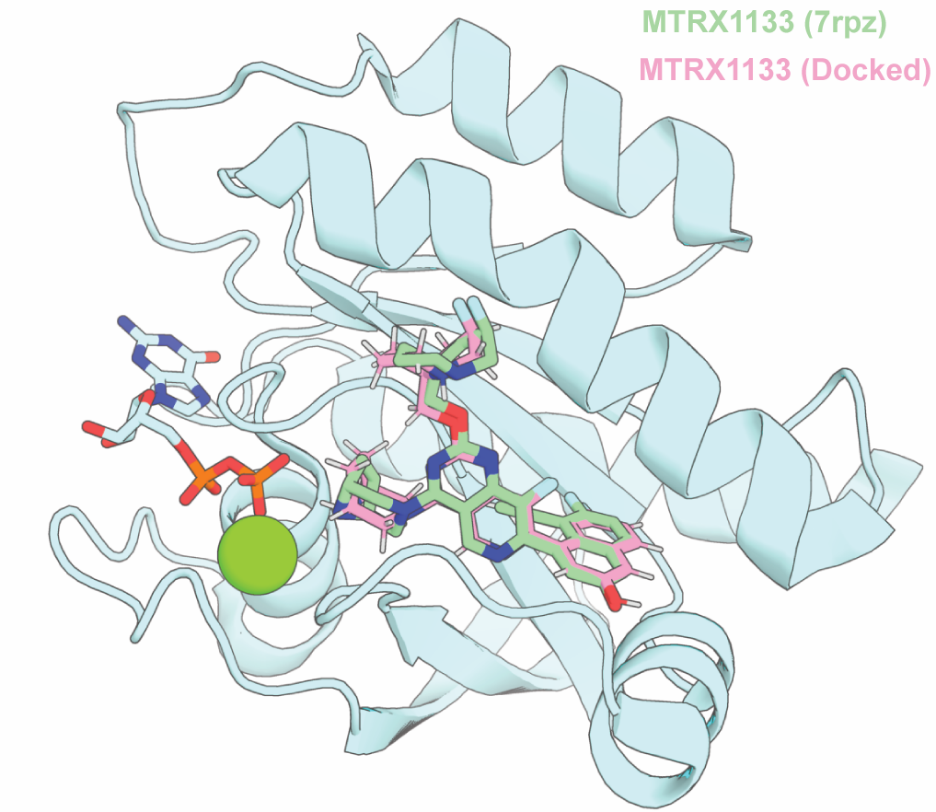
****Figure S1. Overlay of docked and experimental MRTX-1133 ligand structures.** Structural overlay of the computationally docked pose and the crystallographically determined position of the MRTX-1133 ligand. The docked pose closely aligns with the experimental structure with an RMSD of less than 1.0 Å, demonstrating the accuracy and reliability of the docking protocol used in this study.

**
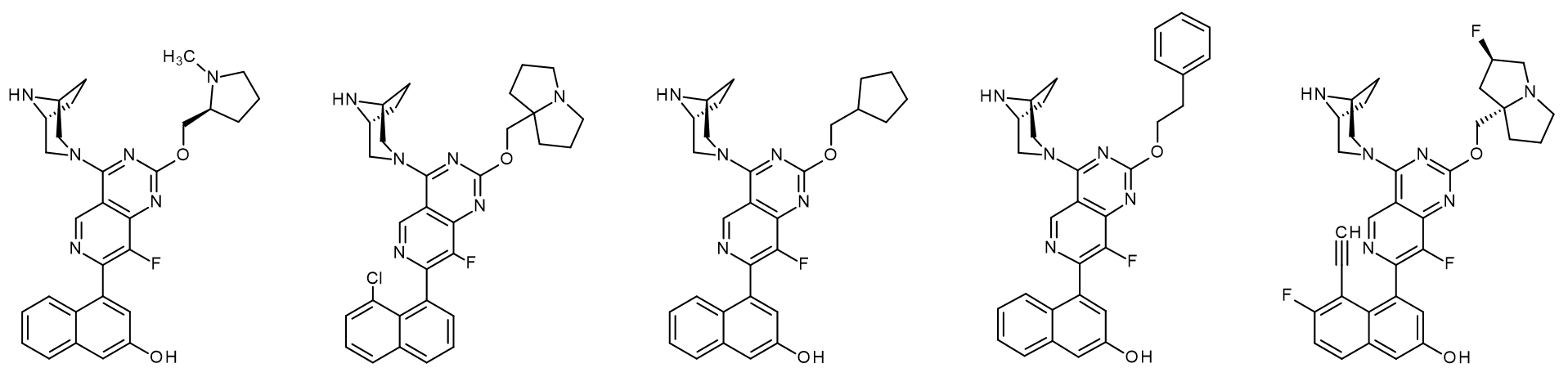
**

**A**

1 2 3 4 5, MRTX1133

**B**

| Compound | P0, nM | L0, nM | Kd, nM | PL, nM | Pf, nM | Pf, % |
| --- | --- | --- | --- | --- | --- | --- |
| 1 | **2000** | **20000** | **100** | 1988.96 | 11.04 | 0.55 |
| 2 | **2000** | **20000** | **5.5** | 1999.39 | 0.61 | 0.03 |
| 3 | **2000** | **20000** | **573** | 1938.50 | 61.50 | 3.07 |
| 4 | **2000** | **20000** | **710** | 1924.41 | 75.59 | 3.78 |
| 5, MRTX1133 | **2000** | **20000** | **0.0002** | 2000.00 | 0.00 | 0.00 |
|  |  |  |  |  |  |  |

**C**

**
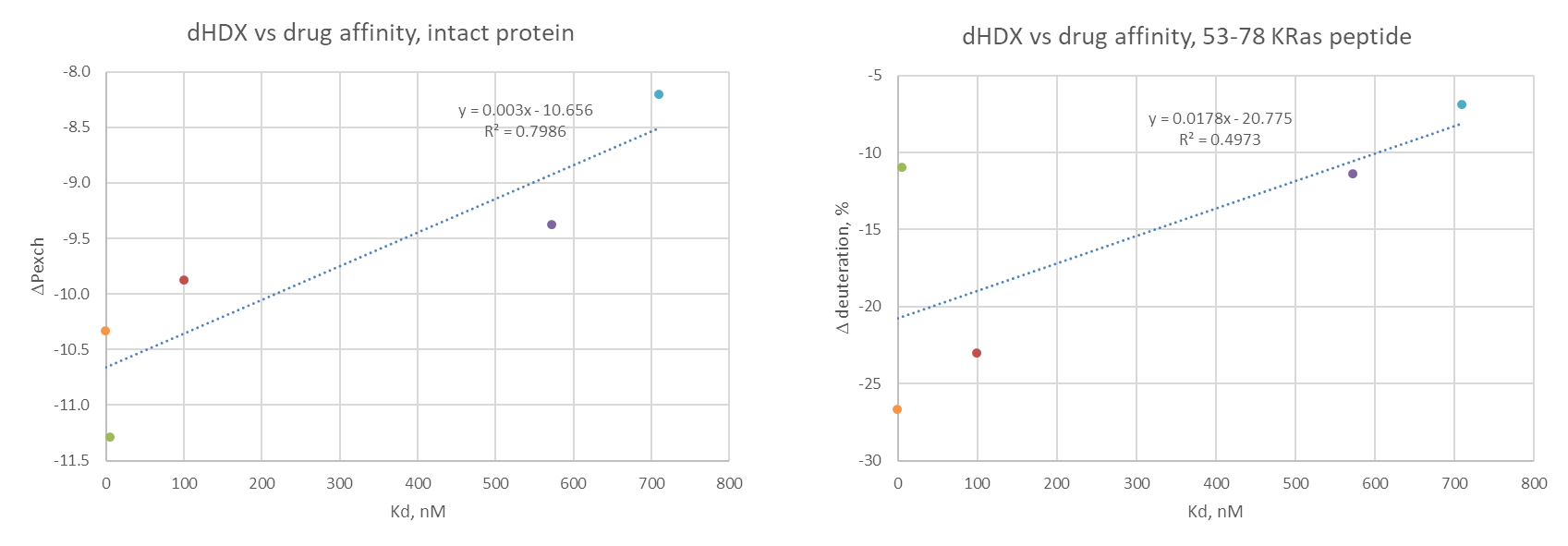
**

**Figure S2. Compounds used in the study. A.** Chemical structure of the compounds used in this study. **B.** Theoretical concentration of the free and bound forms of the KRas G12D protein in the HDX reaction mixtures. P0 – total protein concentration; L0 - total ligand concentration; PL – concentration of the ligand-protein complex; Pf, nM – free protein concentration; Pf, % free protein. The amounts of free protein in the reaction mixtures cannot explain the observed differences in HDX. **C.** Correlation of the observed changes in HDX protection upon protein-ligand complex formation with binding affinities of the compounds.

**
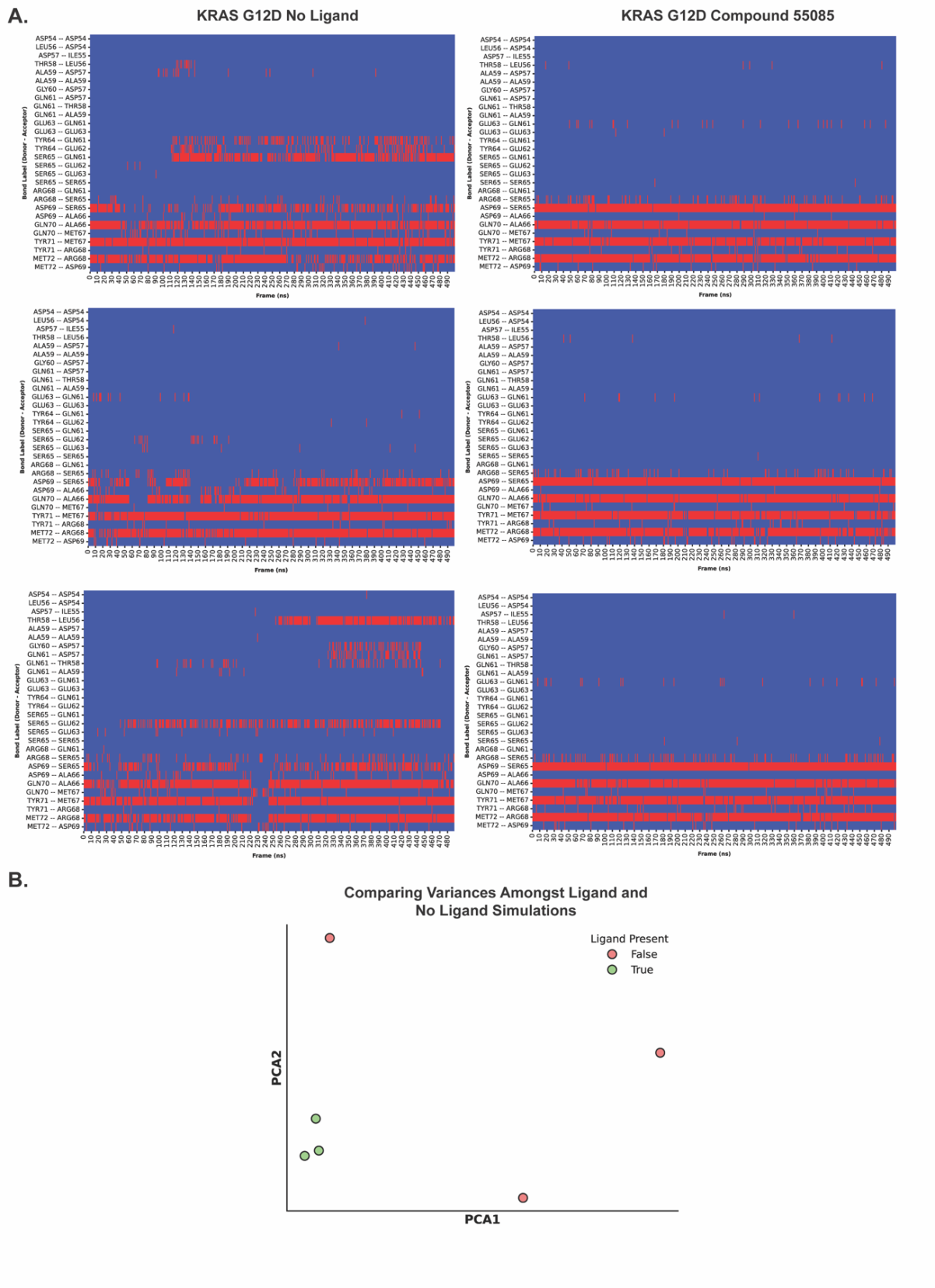
**

**Figure S3. Hydrogen bonding status of the backbone amides in the KRas G12D switch II region in the ligand-free and ligand-bound states.** H-bonds from the MD simulation trajectories for the ligand-free (left panel) and MTRX-1133-bound (right panel) states in switch II region (50 - 70) are shown.

**
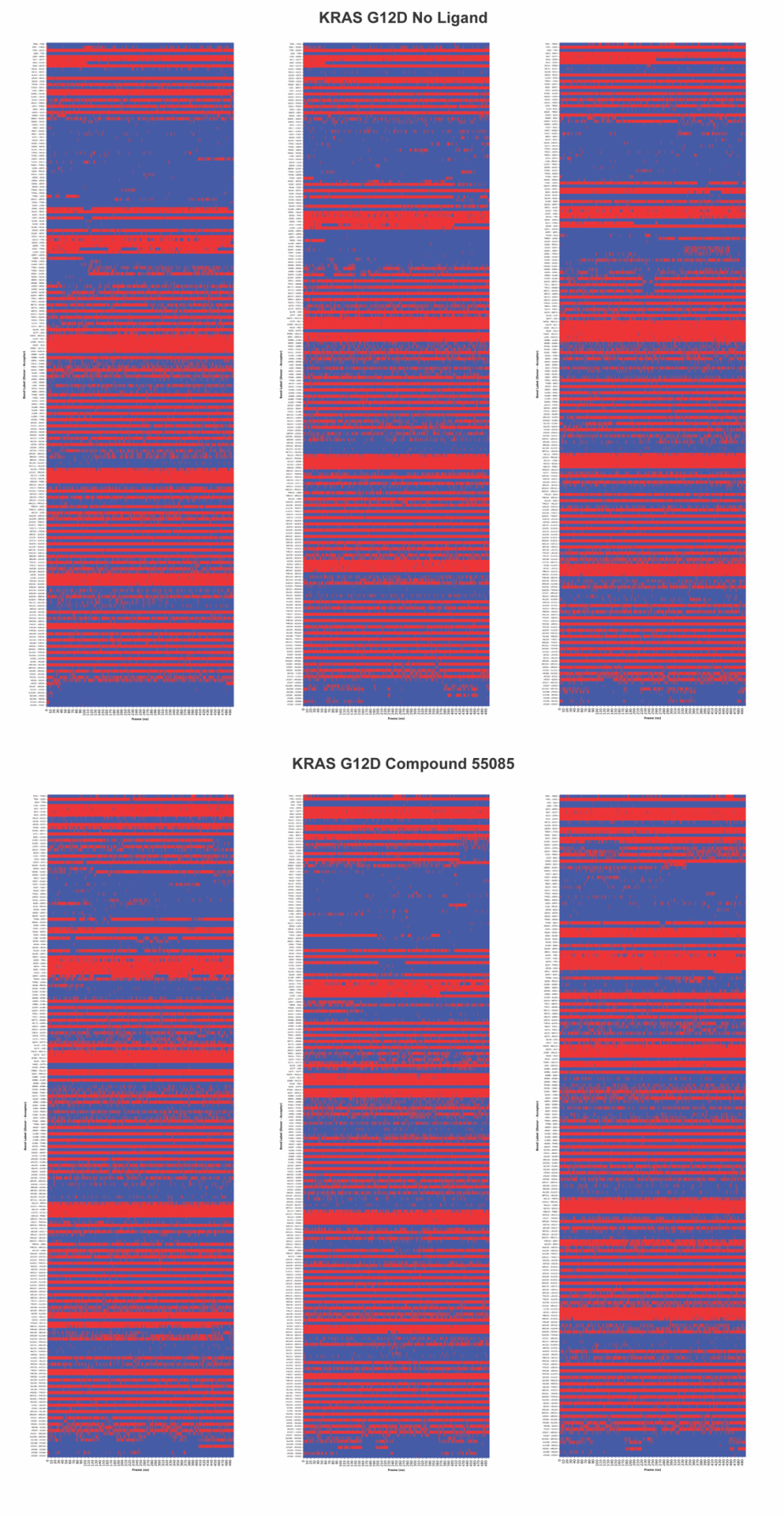
**

**
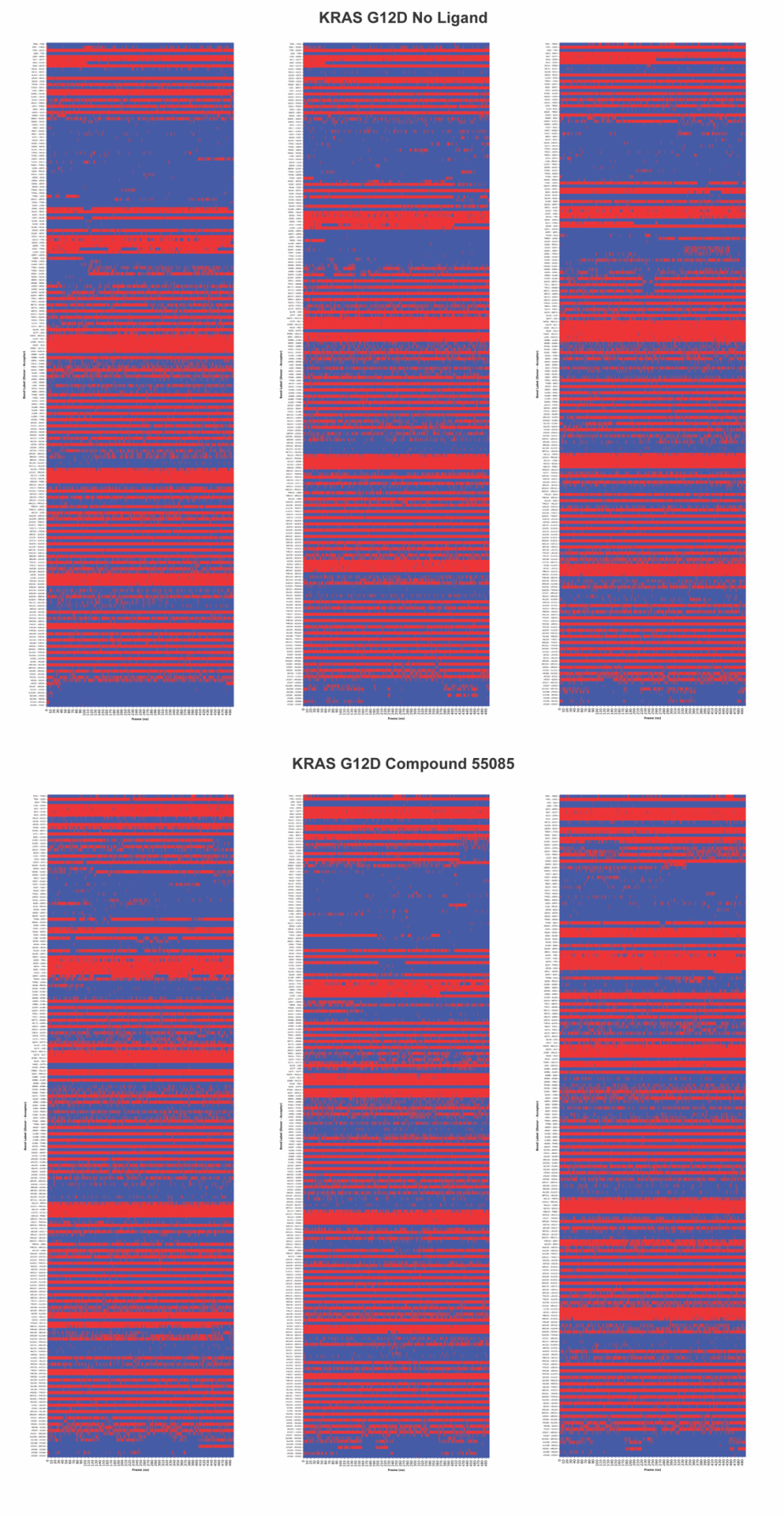
Figure S4. Hydrogen bonding status of the backbone amides of the KRas G12D protein in free and ligand-bound states.** H-bond occupancies from MD simulation trajectories for ligand-free (top panel) and MRTX-1133-bound (bottom panel) states are shown.
